# Thyroid Hormones in Relation to Lead, Mercury, and Cadmium Exposure in the National Health and Nutrition Examination Survey, 2007–2008

**DOI:** 10.1289/ehp.1205239

**Published:** 2012-11-16

**Authors:** Aimin Chen, Stephani S. Kim, Ethan Chung, Kim N. Dietrich

**Affiliations:** Department of Environmental Health, Division of Epidemiology and Biostatistics, University of Cincinnati College of Medicine, Cincinnati, Ohio, USA

**Keywords:** cadmium, heavy metals, lead, mercury, thyroid hormones

## Abstract

Background: Heavy metals, such as lead (Pb), mercury (Hg), and cadmium (Cd), are known toxicants, but their associations with the thyroid axis have not been well quantified at U.S. background levels.

Objectives: We investigated the relationships between thyroid hormones (total and free thyroxine [TT_4_ and FT_4_], total and free triiodothyronine [TT_3_ and FT_3_], thyroid-stimulating hormone [TSH], and thyroglobulin [Tg]) and levels of Pb, Hg, and Cd in blood and Cd in urine.

Methods: We separately analyzed a sample of 1,109 adolescents (12–19 years of age) and a sample of 4,409 adults from the U.S. National Health and Nutrition Examination Survey (NHANES) 2007–2008. We estimated associations after adjusting for age, sex, race, urinary iodine, body mass index, and serum cotinine.

Results: The geometric mean (GM) levels of blood Pb (BPb), total Hg, and Cd were 0.81 µg/dL, 0.47 µg/L, and 0.21 µg/L in adolescents and 1.43 µg/dL, 0.96 µg/L, and 0.38 µg/L in adults, respectively. The GMs of urinary Cd were 0.07 and 0.25 µg/g creatinine in adolescents and adults, respectively. No consistent pattern of metal and thyroid hormone associations was observed in adolescents. In adults, blood Hg was inversely related to TT_4_, TT_3_, and FT_3_ and urinary Cd was positively associated with TT_4_, TT_3_, FT_3_, and Tg, but there were no associations with Pb. Associations were relatively weak at an individual level, with about 1–4% change in thyroid hormones per interquartile range increase in Hg or Cd.

Conclusions: Our analysis suggests an inverse association between Hg exposure and thyroid hormones, and a positive association between Cd exposure and thyroid hormones in adults.

Thyroid hormones (THs) play a critical role in the functions of nervous, reproductive, and cardiovascular systems in both children and adults (Danzi and Klein 2012; Williams 2008; Yazbeck and Sullivan 2012). The hypothalamus–pituitary–thyroid (HPT) axis regulates thyroid function through thyrotropin releasing hormone, thyroid-stimulating hormone (TSH), and the THs [thyroxine (T_4_) and triiodothyronine (T_3_)]. Circulating T_4_ and T_3_ are mostly bound to thyroxine-binding globulin, transthyretin, and albumin; < 1% is unbound and biologically active. In peripheral tissues, T_4_ is converted to T_3_ by type 1 and type 2 deiodinases; T_3_ in turn binds thyroid receptors α and β and initiates target gene expression (Stathatos 2012). Disruption of TH synthesis, transport, deiodination, and metabolism can result in clinical or subclinical thyroid diseases (Cooper and Biondi 2012). Circulating TSH and THs, even at levels within reference ranges, are significantly associated with effects in neurodevelopment (Ghassabian et al. 2011; Pop et al. 2003), blood pressure (Asvold et al. 2007), cholesterol, triglycerides, and insulin resistance (Roos et al. 2007).

Environmental chemicals might alter TH levels via several mechanisms, including disruption of iodine (I) transport, thyroid peroxidase, TH-binding proteins, hepatic catabolism, deiodinases, and receptor binding (Miller et al. 2009). Studies of human populations have focused primarily on chemicals that are structurally similar to T_4_, such as polychlorinated biphenyls (PCBs), polybrominated diphenyl ethers, and bisphenol A (BPA), with little attention on heavy metals (Boas et al. 2006; Pearce and Braverman 2009). Lead (Pb), mercury (Hg), and cadmium (Cd) are known environmental toxicants, but only a few studies have examined associations with total and free T_4_ (TT_4,_ FT_4_), total and free T_3_ (TT_3,_ FT_3_), or TSH (Dundar et al. 2006; Jin et al. 2006; Lamb et al. 2008; Pearce and Braverman 2009; Robins et al. 1983; Schell et al. 2008).

Pb is known to have adverse neurological, hematological, renal, and gastrointestinal effects (Bellinger 2004; Gurer-Orhan et al. 2004); however, associations with THs have been inconsistent (Meeker et al. 2009). Pb exposure [mean 15 µg/dL blood Pb (BPb) level] was negatively correlated with transthyretin levels in cerebrospinal fluid samples from human patients (Zheng et al. 2001). Previous studies of populations with high exposure to Pb (indicated by BPb levels of > 20 µg/dL) suggested negative associations with circulating T_4_, FT_4_, or T_3_ (López et al. 2000; Robins et al. 1983; Singh et al. 2000; Tuppurainen et al. 1988); however, associations were not evident in other similar study populations (Erfurth et al. 2001; Schumacher et al. 1998; Siegel et al. 1989). Fewer studies have investigated associations of BPb levels of < 10 µg/dL with THs. Dundar and colleagues reported a negative association between BPb and FT_4_ levels in adolescents with mean BPb of 7 µg/dL (Dundar et al. 2006). A recent study (Meeker et al. 2009) has suggested an inverse association between BPb (median, 1.5 µg/dL) and TSH levels in men of the couples presenting at infertility clinics. Another study, in the lakeside communities of Quebec, Canada, found no association between BPb (median, 3.1 µg/dL) and THs in men, but identified a positive association with T_3_ and an inverse association with TSH in females with median BPb of 1.7 µg/dL (Abdelouahab et al. 2008).

Hg has adverse effects on a variety of systems that vary with the level, length of exposure, and time window of exposure (Tan et al. 2009). Proposed mechanisms of Hg-related TH disruption involve selective binding to sulfhydryl (SH)-containing ligands in the thyroid, reduced TSH production, and inhibition of deiodination (Soldin et al. 2008; Tan et al. 2009). FT_3_ levels were reduced in association with occupational exposure to Hg vapor among chloralkali plant workers (Barregard et al. 1994; Ellingsen et al. 2000). Studies of populations with environmental exposure, for example, from fish consumption and from dental amalgams, have had mixed findings (Abdelouahab et al. 2008; Meeker et al. 2009; Schell et al. 2008; Takser et al. 2005). A study in a Canadian lakeside community with exposure levels slightly higher than reported for the U.S. National Health and Nutrition Examination Survey (NHANES) [median total Hg, 2.25 µg/L in men and 1.50 µg/L in women compared with median total Hg, 0.8 µg/L in both men and women for NHANES 2003–2006 (Caldwell et al. 2009)] suggested a positive association between Hg and TSH in men only, and no associations with TT_3_ and TT_4_ (Abdelouahab et al. 2008). Hg was not associated with TSH or FT_4_ in 232 Akwesasne Mohawk adolescents with a geometric mean (GM) Hg level of 1 µg/L (Schell et al. 2008).

Cd affects the renal, skeletal, and respiratory systems and is classified as a Group 1 carcinogen by the International Agency for Research on Cancer (Järup and Åkesson 2009). Cd exposure in animal studies has been related to decreased serum TT_4_ levels, and interference with deiodination has been suggested as a possible mechanism (Hammouda et al. 2008; Mori et al. 2006). A Japanese study comparing residents of the Cd-polluted Kakehashi River basin with residents of a nonpolluted area reported lower FT_4_ levels in exposed females but higher TT_3_ levels in both sexes (Nishijo et al. 1994). Studies of neonates and children with environmental exposures have reported inconsistent results (Iijima et al. 2007; Maervoet et al. 2007; Osius et al. 1999). Blood Cd (median, 0.2 µg/L) was not associated with TSH in male infertility clinic patients (Meeker et al. 2009).

Many previous studies have had fairly small sample sizes or have been based on populations with occupational exposures that may not be relevant to the general public. In addition, many studies have measured blood Cd, which is a good biomarker for recent exposure, but urinary Cd is a better indicator of long-term exposure (Järup and Åkesson 2009). In this study, we analyzed NHANES data from 2007–2008 to estimate associations of Pb, Hg, and Cd with TH levels in a large U.S. population with background levels of exposure.

## Methods

We used study subjects’ data from NHANES 2007–2008 [National Center for Health Statistics (NCHS) 2009a] to examine the association between heavy metals and TH levels in the general population with environmental exposure levels. NHANES is conducted in a nationally representative sample of the U.S. civilian population by the Centers for Disease Control and Prevention (CDC; Atlanta, GA). In 2007–2008, a sample of 10,149 subjects was included in this complex multistage, stratified cluster survey. Of those participants, TH levels were measured in 6,260 subjects ≥ 12 years of age. We excluded subjects with no BPb, Hg, or Cd measures (*n* = 5), those who had been told by a doctor or health professional that they have thyroid problems or were currently taking thyroid medications (*n* = 520) (Belin et al. 2004), and those currently pregnant or taking steroid hormones (i.e, estrogen, androgen) that might alter TH or thyroxine-binding globulin levels (*n* = 317). The analytical sample for this analysis was 5,418, including 1,009 adolescents (12–19 years of age) and 4,409 adults (20–80 years of age). After consideration of sampling weights, this analytic sample represents 26,770,162 adolescents and 159,282,838 adults in the general U.S. population who had no reported thyroid diseases, thyroid medications, pregnancy, and sex steroid medications. The analysis was exempt from review by the University of Cincinnati Institutional Review Board, but each subject had provided written informed consent to participate in the NHANES study.

*Heavy metals.* In the NHANES 2007–2008 cycle, metal assays were conducted in whole blood or urine samples at the Division of Laboratory Sciences, National Center for Environmental Health of the CDC. BPb, total Hg, and Cd levels were determined by inductively coupled plasma mass spectrometry (ICP-MS; CDC method no. ITB0001A) with modification from a published method (Nixon et al. 1999) with limits of detection (LOD) of 0.25 µg/dL for Pb, 0.33 µg/L for total Hg, and 0.2 µg/L for Cd (NCHS 2009d). Inorganic Hg in whole blood was measured using Flow Injection Mercury System Cold Vapor Atomic Absorption (PerkinElmer, Norwalk, CT), with an LOD of 0.35 µg/L. In the data set provided by the CDC (2009), levels *<* LOD were imputed as being the metal-specific LOD divided by the square root of 2 (Hornung and Reed 1990).

Only 6 participants had BPb levels *<* LOD. In the United States, organic Hg accounts for the majority of total blood Hg (Mahaffey et al. 2004). Therefore, if the total Hg level was < LOD (*n* = 884), we assumed that organic Hg was equal to the imputed total Hg level (0.2 µg/L). If the total Hg level was > LOD, we calculated organic Hg as the difference between total and inorganic Hg. In this data set, 4,062 subjects (75%) had inorganic Hg levels < LOD; therefore, we did not test for associations between inorganic Hg and TH levels. Blood Cd levels were < LOD in 1,282 subjects. In addition to whole blood samples, a one-third-sample subset of participants in the NHANES 2007–2008 had urine samples tested for Cd (*n* = 1,767) using ICP-MS. Among them, 106 had urine Cd levels < LOD (*<* 0.042 µg/L). We calculated creatinine-adjusted urinary Cd levels to account for urine dilution.

*TH levels.* Serum TH and thyroid antibody levels were determined in the Department of Laboratory Medicine at the University of Washington (Seattle, WA) (NCHS 2009b). Access HYPERsensitive hTSH assay (Beckman Coulter Inc., Brea, CA) was used to assay TSH. Competitive binding immunoenzymatic assay was used to determine TT_4_, FT_4_, TT_3_, and FT_3_. In addition, NHANES 2007–2008 samples were assayed for thyroglobulin (Tg), Tg antibody (TgAb), and thyroid peroxidase antibody (TPOAb) using immunoenzymatic assays.

*Statistical analyses.* In this study, we performed separate analyses for adolescents and adults. In samples from both adolescent (12–19 years of age) and adult subjects (≥ 20 years of age), we first examined the association between heavy metals and TH levels using linear regression models. Because both the exposure and outcome variables were not normally distributed, we used natural log transformation to analyze the data. We examined associations of BPb, blood total Hg, blood organic Hg, blood Cd, and urinary Cd with TT_4_, FT_4_, TT_3_, FT_3_, TSH, and Tg, using separate regression models for each exposure–outcome association. Second, we categorized exposures into quintiles and estimated differences in mean values for the 2nd, 3rd, 4th, and 5th quintiles compared with the first quintile. Third, we examined the proportion of subjects with high levels of TgAb (> 4 IU/mL) or TPOAb (> 9 IU/mL), an indicator of immunologic disturbance of thyroid tissue functions, based on NHANES laboratory method references (NCHS 2009c). Logistic regression was used to estimate odds ratios (ORs) and 95% confidence intervals (CIs) for metal exposure. Because this analytical sample was thyroid disease–free and medication-free (based on self-report) and the percentage of subjects with clinical and subclinical hyperthyroidism or hypothyroidism was < 2% according to NHANES reference values (TSH 0.34–5.6 µIU/mL) (NCHS 2009c), we did not examine hyperthyroidism or hypothyroidism as dichotomous outcomes. Fourth, we did a subset analysis restricted to women of reproductive age (15–44 years of age, *n* = 1,095) to examine whether metal exposure levels in nonpregnant U.S. women had a discernible association with TH levels during reproductive age. Fifth, we summarized significant findings in adolescents and adults by calculating percentage change in TH levels with an interquartile range (IQR) increase in metal exposure levels. Sixth and last, we examined associations between TH levels and multiple metal exposures by categorizing adult BPb, total Hg, and Cd by their corresponding medians (1.39 µg/dL, 0.88 µg/L, 0.33 µg/dL) and modeling the eight possible comparison groups, using the group with levels of all three metals < median as the reference group.

In the regression models, we adjusted for *a priori* covariates (Caldwell et al. 2009; Hollowell et al. 2002; Muntner et al. 2005; Tellez-Plaza et al. 2012): age (continuous), sex (male, female), race/ethnicity (white, black, Hispanic/other), natural log transformed creatinine–adjusted urinary iodine (measured by ICP-MS at the CDC), body mass index (BMI; age- and sex-specific *z*-score in adolescent models, original value in adult models), and serum cotinine levels [measured by high performance liquid chromatography tandem mass spectrometry at the CDC; < 1 ng/mL as nonsmoking, 1–9.9 ng/mL as environmental tobacco smoke (ETS) exposure, ≥ 10 ng/mL as active smoking, dummy variables used] (CDC 2009). Because NHANES is a complex multistage sampling survey, we used PROC SURVEYREG and PROC SURVEYLOGISTIC in SAS version 9.2 (SAS Institute Inc., Cary, NC) to calculate regression parameters and 95% CIs after accounting for sampling weights and survey methods. The significance level was set at α = 0.05 for two-sided tests.

## Results

In the adolescent subjects sample, the mean age was 15.5 years, with 55% male, 60% white, 15% black, and 25% Hispanic and other ethnicity. Twelve percent were exposed to ETS, and 15% were active smokers. The mean BMI *z*-score was 0.54. The GM of urinary iodine was 140 µg/g creatinine. For the adult subjects sample, the mean age was 46.4 years, with 55% male, 68% white, 11% black, and 22% Hispanic and other ethnicity. Adults had 28% active smoking percentage, with 5% exposed to ETS. The mean adult BMI was 28.5 kg/m^2^. The GM of adult urinary iodine was 156 µg/g creatinine. The covariates used in adjusted regression models, including age, sex, race and ethnicity, smoking status, BMI, urinary iodine, were associated with metal exposures and TH levels in most models (detailed data not shown). We also observed increased Pb and Hg levels with age, higher Cd levels in smokers versus nonsmokers and in females versus males, and lower TSH levels in smokers overall.

Table 1 displays the means, ranges, and GMs for Pb, Hg, Cd, and TH levels in both adolescents and adults. Adults had statistically significant higher levels of metal exposures than adolescents.

**Table 1:** Blood and urinary Pb, Hg, and Cd levels and TH levels in the NHANES 2007–2008.

	Adolescents				Adults			
	n	Mean	Range	GM	n	Mean	Range	GM
Metal
Blood Pb (µg/dL)	1,009	0.93	0.18–9.20	0.81	4,409	1.75	0.18–33.1	1.43
Total Hg (µg/L)	1,009	0.68	0.20–6.98	0.47	4,409	1.62	0.20–43.9	0.96
Organic Hg (µg/L)	1,005	0.49	0.01–6.73	0.21	4,404	1.31	0.01–42.9	0.61
Blood Cd (µg/L)	1,009	0.29	0.14–4.70	0.21	4,409	0.55	0.14–8.81	0.38
Urinary Cd (µg/g creatinine)	312	0.08	0.01–1.60	0.07	1,455	0.34	0.02–4.04	0.25
TH
TT4 (µg/dL)	1,009	7.46	1.50–18.50	7.34	4,409	7.63	2.00–27.6	7.48
FT4 (ng/dL)	1,009	0.79	0.10–1.50	0.78	4,409	0.77	0.30–4.80	0.76
TT3 (ng/dL)	1,009	130.37	74.00–241.00	128.36	4,409	112.50	37.0–632.0	110.4
FT3 (pg/mL)	1,009	3.64	2.20–6.00	3.61	4,409	3.21	1.90–20.70	3.18
TSH (µIU/mL)	1,009	1.84	0.01–280.76	1.47	4,409	2.01	0.002–80.97	1.60
Tg (ng/mL)	1,009	10.83	0.07–353.56	7.64	4,409	15.62	0.07–4461.00	9.56
GM, geometric mean.									

**Figure 1:**
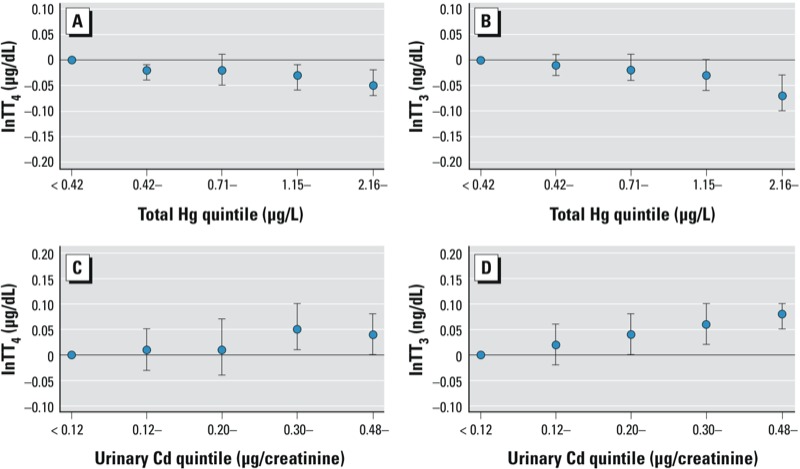
Estimated thyroid hormone levels according to natural log blood total Hg or urinary Cd exposure quintiles in adults, NHANES 2007–2008. (*A*) TT_4_ by blood total Hg quintiles, (*B*) TT_3_ by blood total Hg quintiles, (*C*) TT_4_ by urinary Cd quintiles, (*D*) TT_3_ by urinary Cd quintiles.

Statistically significant negative associations between blood total Hg and TT_4_ and FT_3_ were observed in adolescents (Table 2). Blood Cd was positively associated with FT_3_ and urinary Cd was positively associated with FT_4_. Table 3 shows that in adults, BPb exposure was not associated with any TH levels. Both total and organic Hg had significant negative associations with TT_4_, TT_3_, and FT_3_. Blood Cd was positively associated with FT_4_ and Tg; urinary Cd was positively associated with TT_4_, TT_3_, FT_3_, and Tg. In the analyses of quintiles of metal exposures and TH levels in adolescents, the dose responses were not evident [see Supplemental Material, Table S1 (http://dx.doi.org/10.1289/ehp.1205239)]. However, in adults, the dose–response patterns consistent with the modeling of continuous exposure were evident for total Hg, organic Hg, and urinary Cd (see Supplemental Material, Table S2). Figure 1 shows that total blood Hg levels were associated with lower TT_4_ and TT_3_, with the 5th quintile of exposure (≥ 2.16 µg/dL) showing the strongest associations. Urinary Cd levels were positively associated with TT_4_ and TT_3_. In contrast, no consistent patterns were found for BPb levels (see Supplemental Material, Table S2).

**Table 2:** Adjusted regression coefficients (95% CIs) of blood and urinary Pb, Hg, and Cd in relation to THs in adolescents.

Metal	lnTT4	lnFT4	lnTT3	lnFT3	lnTSH	lnTg
lnBPb	0.01 (–0.02, 0.04)	0.01 (–0.01, 0.04)	0.01 (–0.01, 0.04)	0.02 (–0.002, 0.04)	–0.05 (–0.18, 0.07)	0.05 (–0.13, 0.24)
lnTotal Hg	–0.02 (–0.04, –0.001)*	0.005 (–0.01, 0.02)	–0.02 (–0.03, 0.001)	–0.01 (–0.02, –0.003)*	0.02 (–0.04, 0.08)	–0.05 (–0.20, 0.10)
lnOrganic Hg	–0.01 (–0.03, 0.004)	0.01 (–0.01, 0.02)	–0.01 (–0.02, 0.01)	–0.01 (–0.02, –0.001)*	–0.01 (–0.06, 0.04)	–0.06 (–0.17, 0.04)
lnBlood Cd	–0.01 (–0.06, 0.03)	–0.01 (–0.05, 0.04)	–0.01 (–0.03, 0.02)	0.02 (0.0003, 0.03)*	–0.11 (–0.27, 0.04)	0.07 (–0.15, 0.28)
lnUrinary Cd	–0.0003 (–0.03, 0.03)	0.04 (0.002, 0.08)*	0.02 (–0.02, 0.06)	0.02 (–0.01, 0.04)	–0.03 (–0.24, 0.17)	0.08 (–0.13, 0.30)
Adjusted for age, sex, race/ethnicity, creatinine-adjusted urinary iodine, BMI z–score, and serum cotinine level. *p < 0.05.						

**Table 3:** Adjusted regression coefficients (95% CIs) of blood and urinary Pb, Hg, and Cd in relation to THs in adults.

Metal	lnTT4	lnFT4	lnTT3	lnFT3	lnTSH	lnTg
lnBPb	–0.01 (–0.02, 0.01)	0.01 (–0.01, 0.02)	–0.0004 (–0.02, 0.02)	0.01 (–0.001, 0.02)	–0.01 (–0.06, 0.04)	0.01 (–0.03, 0.06)
lnTotal Hg	–0.02 (–0.02, –0.01)*	–0.005 (–0.01, 0.004)	–0.03 (–0.04, –0.01)*	–0.01 (–0.02, –0.01)*	0.004 (–0.02, 0.03)	–0.01 (–0.05, 0.03)
lnOrganic Hg	–0.01 (–0.02, –0.004)*	–0.004 (–0.01, 0.001)	–0.02 (–0.03, –0.01)*	–0.01 (–0.01, –0.004)*	0.01 (–0.01, 0.03)	0.001 (–0.02, 0.03)
lnBlood Cd	0.005 (–0.01, 0.02)	0.01 (0.001, 0.02)*	0.004 (–0.01, 0.02)	0.0002 (–0.01, 0.01)	–0.02 (–0.07, 0.03)	0.10 (0.05, 0.16)*
lnUrinary Cd	0.02 (0.001, 0.03)*	0.01 (–0.01, 0.03)	0.03 (0.02, 0.05)*	0.01 (0.002, 0.02)*	–0.04 (–0.09, 0.02)	0.15 (0.23, 0.25)*
Adjusted for age, sex, race/ethnicity, creatinine-adjusted urinary iodine, BMI value, and serum cotinine level. *p < 0.05.						

The percentages of high thyroid antibody levels were slightly higher in adults than adolescents (5.77% vs. 4.79% for TgAb; 8.83% vs. 6.04% for TPOAb, Table 4). However, in neither adolescents nor adults were blood or urinary Pb, Hg, Cd levels significantly associated with high TgAb or TPOAb levels.

**Table 4:** Adjusted ORs (95% CIs) of high TgAb (> 4 IU/mL) and high TPOAb (> 9 IU/mL) per unit change in ln metal exposure in adolescents and adults.

Metal	Adolescentsa		Adultsb	
	High TgAb	High TPOAb	High TgAb	High TPOAb
Percent	4.79	6.04	5.77	8.83
lnBPb	0.77 (0.37–1.59)	1.16 (0.58–2.33)	1.20 (0.87–1.64)	1.09 (0.82–1.46)
lnTotal Hg	1.17 (0.64–2.13)	1.15 (0.68–1.95)	0.94 (0.78–1.13)	0.91 (0.80–1.04)
lnOrganic Hg	1.29 (0.85–1.96)	1.06 (0.66–1.72)	0.95 (0.81–1.11)	0.96 (0.87–1.06)
lnBlood Cd	1.18 (0.56–2.51)	1.44 (0.71–2.95)	1.09 (0.74–1.60)	1.14 (0.85–1.51)
lnUrinary Cd	0.99 (0.49–2.02)	0.97 (0.41–2.28)	0.84 (0.47–1.48)	1.20 (0.75–1.93)
aAdjusted for age, sex, race/ethnicity, creatinine-adjusted urinary iodine, BMI z-score, and serum cotinine level. bAdjusted for age, sex, race/ethnicity, creatinine-adjusted urinary iodine, BMI value, and serum cotinine level.					

Subset analyses of continuous exposures among women of reproductive age were generally consistent with associations in the adult population as a whole [see Supplemental Material Table S3 (http://dx.doi.org/10.1289/ehp.1205239)]. Urinary Cd levels were positively related to TT_4_, but associations with other TH levels did not reach statistical significance.

Table 5 gives the estimated percentage difference in TH levels per IQR increase in exposures that were significantly associated with TH levels when modeled as continuous variables. Overall estimated differences in mean levels were small, at 1–4%. However, we estimated a 12% increase in Tg associated with blood Cd at 0.61 vs. 0.21 µg/L, and an 18% increase in Tg associated with urinary Cd of 0.41 vs. 0.14 µg/g creatinine in adults.

**Table 5:** Interpretation of the observed association per IQR change in exposure on TH levels.

Population/metal exposure	IQR (P25 to P75) or LOD/√–2a to P75	lnP75–lnP25	Association with THs	Regression estimate for IQR change (95% CI)	Percentage difference in THs (95% CI) for exposure at P75 compared with P25 or LOD/√–2 if > 25% of subjects had exposure < LOD
Adolescents
Total Hg (µg/L)	0.20a–0.82	1.41	↓TT4	–0.03 (–0.05, –0.001)	–2.6 (–5.0, –0.1)
			↓FT3	–0.02 (–0.03, –0.004)	–1.6 (–2.7, –0.4)
Organic Hg (µg/L)	0.20a–0.53	0.97	↓FT3	–0.01 (–0.02, –0.001)	–0.8 (–1.6, –0.1)
Blood Cd (µg/L)	0.14a–0.25	0.58	↑FT3	0.01 (0.0001, 0.02)	0.9 (0.01, 1.9)
Urinary Cd (µg/g creatinine)	0.04–0.11	1.01	↑FT4	0.04 (0.002, 0.08)	4.3 (0.2, 8.5)
Adults
Total Hg (µg/L)	0.49–1.80	1.30	↓TT4	–0.02 (–0.03, –0.008)	–2.0 (–3.0, –0.8)
			↓TT3	–0.03 (–0.05, –0.02)	–3.3 (–5.0, –1.5)
			↓FT3	–0.02 (–0.02, –0.01)	–1.7 (–2.2, –1.2)
Organic Hg (µg/L)	0.21–1.39	1.89	↓TT4	–0.02 (–0.03, –0.01)	–2.0 (–3.2, –0.8)
			↓TT3	–0.03 (–0.05, –0.01)	–3.2 (–5.2, –1.1)
			↓FT3	–0.01 (–0.02, –0.01)	–1.4 (–2.1, –0.7)
Blood Cd (µg/L)	0.21–0.61	1.07	↑FT4	0.01 (0.001, 0.02)	1.0 (0.1, 1.9)
			↑Tg	0.11 (0.06, 0.17)	11.9 (6.0, 18.1)
Urinary Cd (µg/g creatinine)	0.14–0.41	1.07	↑TT4	0.02 (0.001, 0.03)	1.9 (0.1, 3.6)
			↑TT3	0.04 (0.02, 0.05)	3.6 (1.8, 5.4)
			↑FT3	0.01 (0.002, 0.02)	1.0 (0.2, 1.9)
			↑Tg	0.16 (0.07, 0.25)	17.5 (7.7, 28.3)
Abbreviations: P75, 75th percentile; P25, 25th percentile. aInstead of first quartile, the interval starts from < LOD (value replaced with LOD divided by the square root of 2) to reflect > 25% participants with exposure < LOD.					

In the three-metal analysis in adults, the negative association between total Hg and TT_4_ and TT_3_ was evident with and without exposures to Cd or Pb above median levels, and the positive association between blood Cd and Tg was evident for all combinations with exposure to Cd above the median, regardless of exposure to Hg or Pb [see Supplemental Material, Table S4 (http://dx.doi.org/10.1289/ehp.1205239)].

## Discussion

In adults, Hg exposure was negatively associated with TH levels, whereas Cd exposure was positively associated with TH levels and the pre-hormone Tg. TSH levels were not consistently associated with Hg or Cd exposure, suggesting that these exposures may not affect pituitary function. In women of reproductive age, the inverse associations between Hg and TH levels persisted, whereas the associations between Cd and TH levels were mostly positive but not statistically significant.

The lack of association between BPb and TH levels suggests current exposure levels experienced by the U.S. population do not adversely affect TH synthesis and regulation, though effects at higher environmental exposure levels cannot be ruled out. Occupational Pb exposure has been associated with significant reductions in TH levels (Robins et al. 1983), and a recent animal study also noted an effect of Pb on TH levels in rats (Wu et al. 2011).

Negative associations observed between Hg and TH levels are consistent with proposed mechanisms for Hg toxicity in which Hg accumulates in the thyroid and reduces iodide uptake at the sodium/iodide symporter by binding to iodide (Nishida et al. 1986) and inhibits TH deiodinase function in peripheral tissues (Soldin et al. 2008; Tan et al. 2009). In the Abdelouahab et al. (2008) study, TSH was positively associated with hair and blood Hgs, but we did not find an association between TSH and blood Hg in our population. A recent analysis of NHANES 2007–2008 data suggested an increase in the prevalence of TgAb in women with blood Hg > 1.8 µg/L versus ≤ 0.4 µg/L (Gallagher and Meliker 2012), but that analysis did not exclude subjects that had thyroid disease or were taking medications to treat thyroid disease. We did not evaluate inorganic Hg, which has been associated with a higher FT_4_/FT_3_ ratio in two occupational studies (Barregard et al. 1994; Ellingsen et al. 2000). Prior research has suggested that PCBs may influence TH levels, and effects of PCB could therefore confound associations with Hg because both may be consumed in fish (Hagmar 2003). However, we did not have data on PCB exposures.

Studies in experimental animals suggest lower TH levels in Cd-exposed mice and rats (Gupta and Kar 1997, 1998; Hammouda et al. 2008; Yoshizuka et al. 1991), in contrast with our finding of positive associations between Cd and TH levels. This discrepancy could be due to species differences or to higher exposure doses being used in animal studies, although we cannot rule out the possibility that the associations we observed were due to chance or bias. TT_3_ was increased in residents of a Cd-polluted region (GM urinary Cd levels, 6.6 and 9.2 µg/g creatinine in males and females, respectively) compared with TT_3_ levels in residents of a control region (GMs of 2.6 and 4.4 µg/g creatinine, respectively) (Nishijo et al. 1994). In the present study population, GM urinary Cd levels were an order of magnitude lower, but we observed higher TH levels with increased urinary Cd. Additional research is needed to clarify the potential effects of Cd exposure on thyroid function in humans.

The observed associations between Hg and Cd exposures and TH levels were relatively weak on the individual level, with an IQR increase in exposure associated with a 1–4% change in hormone levels. The HPT axis is precisely regulated and it is plausible that the low environmental exposures experienced by the U.S. population do not substantially influence individual thyroid profiles. However, exposures at the very high end (e.g., ≥ 5th quintile compared with the 1st quintile) may be associated with a TH level change of > 5%, such as total Hg at ≥ 2.16 µg/L versus < 0.42 µg/L and TT_3_ in adults as shown in Supplemental Material, Table S2 (http://dx.doi.org/10.1289/ehp.1205239). This may be related to significant health effects in individuals who already have compromised thyroid functions. More research is needed for Hg and Cd exposure at the higher end of exposures, for example, in specific populations such as people who eat a large amount of fish or live close to Cd-contaminated areas.

This analysis has limitations related to the cross-sectional design of the NHANES. The associations cannot be interpreted as causation, and the results are more relevant to background exposure in the general population. Although we did analyze levels of BPb, Hg, and Cd as well as urinary Cd, the latter is only available in a subset of one-third of the total sample population. The research was only assessing one time point and we lacked longitudinal data. We performed multiple comparisons in the analysis and may have encountered the problem of false positive findings. Instead of adopting a strict Bonferroni correction, we mainly focused the patterns (relation to more than one TH), dose response (significance for more than one quintile), and consistency between exposures (total and organic Hg, blood and urinary Cd). In spite of these limitations, we were able to test the associations between metals and FT_4_ and FT_3_, which often were not measured in prior studies. We stratified the analysis by adolescents and adults, and replicated results in women of reproductive age. We completed the analysis using continuous exposure variable and exposure quintiles, and we summarized percentage change in hormone levels by IQR of exposure.

## Conclusions

In the general adult U.S. population, we observed inverse associations between Hg and TH levels and positive associations between Cd and TH levels. Research is needed to quantify the associations at higher levels of exposure and to examine potential mechanisms of Hg and Cd thyroid toxicity.

## Supplemental Material

(164 KB) PDF
