# Supplementary material for: Thyroid Hormones in Relation to Lead, Mercury, and Cadmium Exposure in the National Health and Nutrition Examination Survey, 2007–2008
Source: Environ Health Perspect. 2012 Nov 16;121(2):181–6. doi: 10.1289/ehp.1205239 (PMC3569681; doi:10.1289/ehp.1205239)
Supplement: (164 KB) PDF [file ehp.1205239.s001.pdf]

Supplemental Material

Thyroid Hormones in Relation to Lead, Mercury, and Cadmium Exposure in the National Health and Nutrition Examination Survey, 2007–2008

Aimin Chen[1], Stephani S. Kim[1], Ethan Chung[1], Kim N. Dietrich[1]

[1]Division of Epidemiology and Biostatistics, Department of Environmental Health, University of Cincinnati College of Medicine, Cincinnati, Ohio, USA

Table of Contents

Table S1: Adjusted regression coefficients and 95% confidence intervals of blood and urinary lead, mercury, cadmium quintiles in relation to thyroid hormones in adolescents

Table S2: Adjusted regression coefficients and 95% confidence intervals of blood and urinary lead, mercury, cadmium quintiles in relation to thyroid hormones in adults

Table S3: Adjusted regression coefficients and 95% confidence intervals of blood and urinary lead, mercury, cadmium in relation to thyroid hormones in women of reproductive age (15-44 years)

Table S4: Adjusted regression coefficients and 95% confidence intervals of cumulative exposure to lead, mercury, and cadmium in relation to thyroid hormones in adults

Supplemental Material, Table S1. Adjusted regression coefficients and 95% confidence intervals of blood and urinary lead, mercury, cadmium quintiles in relation to thyroid hormones in adolescents[a]

Metal	Quintiles	$lnTT_4$	$lnFT_4$	$lnTT_3$	$lnFT_3$	lnTSH	lnTg
Pb	Q1 <0.53	reference	reference	reference	reference	reference	reference
(μg/dL)	Q2 0.53-	-0.02 (-0.07, 0.04)	0.01 (-0.04, 0.06)	-0.01 (-0.05, 0.03)	0.01 (-0.02, 0.03)	-0.09 (-0.22, 0.05)	0.10 (-0.18, 0.38)
	Q3 0.70-	-0.05 (-0.11, 0.01)	0.003 (-0.04, 0.05)	0.005 (-0.05, 0.04)	0.02 (-0.01, 0.04)	-0.10 (-0.25, 0.05)	-0.01 (-0.36, 0.34)
	Q4 0.87-	-0.02 (-0.09, 0.06)	0.01 (-0.06, 0.07)	0.03 (-0.03, 0.08)	0.03 (0.002, 0.06)*	-0.28 (-0.52, -0.04)*	0.11 (-0.14, 0.37)
	Q5 1.18-	0.002 (-0.06, 0.06)	0.02 (-0.03, 0.06)	0.01 (-0.02, 0.05)	0.03 (-0.0001, 0.06)	-0.12 (-0.29, 0.04)	0.04 (-0.21, 0.30)
Total	<LOD	reference	reference	reference	reference	reference	reference
Hg	Qrt1 0.21-	-0.01 (-0.06, 0.04)	-0.02 (-0.07, 0.02)	0.01 (-0.05, 0.06)	0.01 (-0.02, 0.04)	0.12 (-0.01, 0.25)	0.11 (-0.20, 0.41)
(μg/L)[b]	Qrt2 0.45-	-0.02 (-0.07, 0.02)	0.01 (-0.05, 0.07)	-0.04 (-0.09, 0.01)	-0.01 (-0.04, 0.03)	0.03 (-0.10, 0.17)	-0.28 (-0.55, -0.01)*
	Qrt3 0.66-	-0.04 (-0.09, 0.02)	-0.01 (-0.05, 0.03)	-0.02 (-0.07, 0.03)	-0.02 (-0.04, 0.01)	-0.01 (-0.17, 0.16)	-0.07 (-0.31, 0.18)
	Qrt4 1.12-	-0.02 (-0.06, 0.01)	0.02 (-0.03, 0.07)	-0.03 (-0.06, 0.01)	-0.02 (-0.04, 0.01)	0.06 (-0.06, 0.18)	-0.02 (-0.36, 0.32)
Organic	<LOD	reference	reference	reference	reference	reference	reference
Hg	Qrt1 0.21-	-0.01 (-0.06, 0.03)	0.02 (-0.03, 0.07)	-0.03 (-0.07, 0.01)	-0.02 (-0.06, 0.03)	-0.01 (-0.23, 0.22)	-0.22 (-0.44, -0.01)*
(μg/L)[b]	Qrt2 0.34-	-0.02 (-0.08, 0.05)	-0.01 (-0.06, 0.05)	-0.05 (-0.10, -0.01)*	-0.02 (-0.05, 0.01)	0.02 (-0.09, 0.13)	-0.38 (-0.67, -0.08)*
	Qrt3 0.56-	-0.03 (-0.08, 0.01)	0.003 (-0.03, 0.04)	-0.01 (-0.05, 0.04)	-0.01 (-0.04, 0.02)	-0.17 (-0.21, 0.07)	-0.07 (-0.23, 0.09)
	Qrt4 1.04-	-0.03 (-0.08, 0.01)	0.02 (-0.03, 0.07)	-0.03 (-0.07, 0.00)	-0.03 (-0.05, -0.01)*	0.04 (-0.13, 0.21)	-0.08 (-0.44, 0.28)

Supplemental Material, Table S1 (continued)

Metal	Quintiles	$lnTT_4$	$lnFT_4$	$lnTT_3$	$lnFT_3$	lnTSH	lnTg
Blood	<LOD	reference	reference	reference	reference	reference	reference
Cd	Qrt1 0.15-	-0.03 (-0.08, 0.01)	-0.02 (-0.07, 0.04)	0.004 (-0.02, 0.03)	0.01 (-0.02, 0.03)	-0.06 (-0.20, 0.08)	-0.02 (-0.47, 0.43)
(μg/L)[b]	Qrt2 0.22-	0.02 (-0.04, 0.08)	0.01 (-0.04, 0.06)	0.02 (-0.03, 0.07)	0.01 (-0.03, 0.05)	-0.10 (-0.21, 0.004)	0.09 (-0.08, 0.26)
	Qrt3 0.26-	-0.01 (-0.05, 0.02)	-0.004 (-0.07, 0.06)	0.03 (-0.01, 0.06)	0.02 (-0.01, 0.05)	-0.22 (-0.46, 0.02)	0.11 (-0.18, 0.39)
	Qrt4 0.42-	-0.05 (-0.14, 0.04)	-0.01 (-0.08, 0.07)	-0.02 (-0.08, 0.05)	0.03 (-0.01, 0.07)	-0.07 (-0.40, 0.27)	0.06 (-0.65, 0.52)
Urinary	Q1 <0.04	reference	reference	reference	reference	reference	reference
Cd	Q2 0.04-	0.08 (0.003, 0.15)*	0.07 (-0.001, 0.13)	0.04 (-0.003, 0.07)	0.02 (-0.03, 0.06)	0.03 (-0.25, 0.32)	-0.35 (-0.91, 0.21)
(μg/g	Q3 0.06-	0.05 (-0.03, 0.13)	0.02 (-0.04, 0.07)	0.02 (-0.04, 0.07)	-0.02 (-0.09, 0.04)	-0.16 (-0.42, 0.10)	-0.40 (-0.81, 0.02)
creatini	Q4 0.08-	0.03 (-0.03, 0.11)	0.11 (-0.001, 0.22)	0.04 (-0.02, 0.11)	0.05 (-0.001, 0.10)	-0.28 (-0.74, 0.18)	0.03 (-0.37 0.42)
ne)	Q5 0.11-	0.03 (-0.06, 0.11)	0.06 (-0.001, 0.13)	0.06 (-0.02, 0.14)	0.03 (-0.01, 0.08)	0.07 (-0.28, 0.41)	0.17 (-0.15 0.48)

[a]: Adjusted for age, sex, race/ethnicity, creatinine adjusted urinary iodine, BMI z-score, and serum cotinine concentration

[b]: Instead of quintiles, here the reference group is exposure <LOD, and four comparison groups are categorized using quartiles of values above LOD

*: $p<0.05$

Supplemental Material, Table S2. Adjusted regression coefficients and 95% confidence intervals of blood and urinary lead, mercury, cadmium quintiles in relation to thyroid hormones in adults[a]

Metal	Quintile	$lnTT_4$	$lnFT_4$	$lnTT_3$	$lnFT_3$	lnTSH	lnTg
Pb	Q1 <0.85	reference	reference	reference	reference	reference	reference
(µg/dL)	Q2 0.85-	-0.01 (-0.04, 0.02)	0.02 (-0.01, 0.04)	-0.01 (-0.03, 0.01)	0.002 (-0.02, 0.02)	-0.04 (-0.13, 0.04)	0.07 (-0.03, 0.17)
	Q3 1.19-	-0.01 (-0.04, 0.02)	0.02 (-0.02, 0.05)	0.01 (-0.03, 0.04)	0.01 (-0.01, 0.03)	-0.005 (-0.08, 0.07)	0.05 (-0.07, 0.18)
	Q4 1.60-	0.001 (-0.03, 0.03)	0.03 (0.002, 0.05)*	0.002 (-0.02, 0.03)	0.02 (-0.001, 0.04)	-0.07 (-0.16, 0.03)	0.10 (-0.02, 0.23)
	Q5 2.31-	-0.01 (-0.04, 0.02)	0.02 (-0.01, 0.05)	-0.004 (-0.03, 0.02)	0.02 (-0.001, 0.03)	-0.04 (-0.13, 0.05)	0.04 (-0.06, 0.14)
Total	Q1 <0.42	reference	reference	reference	reference	reference	reference
Hg	Q2 0.42-	-0.02 (-0.04, -0.01)*	0.01 (-0.003, 0.03)	-0.01 (-0.03, 0.01)	-0.01 (-0.03, -0.001)*	-0.03 (-0.11, 0.05)	-0.02 (-0.15, 0.12)
(µg/L)	Q3 0.71-	-0.02 (-0.05, 0.01)	0.002 (-0.02, 0.02)	-0.02 (-0.04, 0.01)	-0.02 (-0.03, -0.004)*	-0.05 (-0.15, 0.05)	0.04 (-0.08, 0.15)
	Q4 1.15-	-0.03 (-0.06, -0.01)*	-0.01 (-0.04, 0.02)	-0.03 (-0.06, -0.00)*	-0.02 (-0.03, -0.01)*	-0.03 (-0.11, 0.05)	0.01 (-0.10, 0.11)
	Q5 2.16-	-0.05 (-0.07, -0.02)*	-0.01 (-0.03, 0.01)	-0.07 (-0.10, -0.03)*	-0.04 (-0.05, -0.03)*	-0.01 (-0.09, 0.07)	-0.01 (-0.14, 0.12)
Organic	Q1 <0.21	reference	reference	reference	reference	reference	reference
Hg	Q2 0.21-	-0.01 (-0.03, 0.01)	0.02 (-0.01, 0.04)	0.00 (-0.03, 0.03)	-0.01 (-0.02, 0.01)	-0.07 (-0.18, 0.03)	-0.02 (-0.19, 0.15)
(µg/L)	Q3 0.42-	-0.02 (-0.04, 0.01)	-0.01 (-0.02, 0.02)	-0.02 (-0.03, 0.004)	-0.02 (-0.03, -0.01)*	-0.02 (-0.11, 0.07)	0.03 (-0.08, 0.13)
	Q4 0.82	-0.03 (-0.06, 0.001)	-0.01 (-0.04, 0.01)	-0.03 (-0.06, 0.001)	-0.02 (-0.03, -0.01)*	-0.01 (-0.07, 0.05)	0.00 (-0.11, 0.12)
	Q5 1.76-	-0.04 (-0.06, -0.02)*	-0.01 (-0.03, 0.01)	-0.07 (-0.10, -0.03)*	-0.03 (-0.04, -0.02)*	0.00 (-0.07, 0.07)	-0.02 (-0.12, 0.08)

Supplemental Material, Table S2 (continued)

Metal	Quintile	$lnTT_4$	$lnFT_4$	$lnTT_3$	$lnFT_3$	lnTSH	lnTg
Blood	Q1 <0.15	reference	reference	reference	reference	reference	reference
Cd	Q2 0.15-	-0.01 (-0.03, 0.02)	0.01 (-0.01, 0.04)	-0.01 (-0.04, 0.01)	0.01 (-0.01, 0.03)	0.06 (0.004, 0.11)*	-0.11 (-0.26, 0.03)
(μg/L)	Q3 0.27-	0.01 (-0.02, 0.04)	0.02 (-0.004, 0.04)	0.01 (-0.02, 0.04)	0.004 (-0.01, 0.02)	0.03 (-0.01, 0.07)*	-0.12 (-0.21, -0.03)
	Q4 0.41-	0.01 (-0.02, 0.03)	0.02 (-0.0002, 0.04)	0.002 (-0.02, 0.03)	0.003 (-0.01, 0.02)	-0.05 (-0.12, 0.03)	0.09 (-0.002, 0.18)
	Q5 0.74-	0.02 (-0.01, 0.04)	0.03 (0.01, 0.05)*	0.005 (-0.04, 0.04)	0.004 (-0.02, 0.03)	-0.05 (-0.17, 0.07)	0.17 (0.04, 0.31)*
Urinary	Q1 <0.12	reference	reference	reference	reference	reference	reference
Cd	Q2 0.12-	0.01 (-0.03, 0.05)	-0.01 (-0.05, 0.03)	0.02 (-0.02, 0.06)	0.02 (0.003, 0.03)*	-0.04 (-0.17, 0.08)	0.04 (-0.16, 0.23)
(μg/g	Q3 0.20-	0.01 (-0.04, 0.07)	-0.01 (-0.06, 0.05)	0.04 (-0.003, 0.08)	0.004 (-0.02, 0.03)	-0.13 (-0.22, -0.04)*	0.14 (-0.02, 0.31)
creatini	Q4 0.30-	0.05 (0.01, 0.10)*	-0.002 (-0.04, 0.04)	0.06 (0.02, 0.10)*	0.03 (0.002, 0.05)*	-0.09 (-0.23, 0.06)	0.25 (-0.02, 0.53)
ne)	Q5 0.48-	0.04 (0.001, 0.08)*	0.02 (-0.02, 0.06)	0.08 (0.05, 0.10)*	0.03 (0.01, 0.05)*	-0.09 (-0.24, 0.06)	0.32 (0.06, 0.58)*

[a]: Adjusted for age, sex, race/ethnicity, creatinine adjusted urinary iodine, BMI value, and serum cotinine concentration

*: $p<0.05$

Supplemental Material, Table S3. Adjusted regression coefficients and 95% confidence intervals of blood and urinary lead, mercury, cadmium in relation to thyroid hormones in women of reproductive age (15-44 years)[a]

Metal	$lnTT_4$	$lnFT_4$	$lnTT_3$	$lnFT_3$	lnTSH	lnTg
ln blood Pb	-0.02 (-0.07, 0.03)	0.02 (-0.03, 0.07)	-0.01 (-0.06, 0.04)	0.01 (-0.03, 0.05)	-0.04 (-0.19, 0.10)	-0.13 (-0.35, 0.09)
ln total Hg	-0.03 (-0.05, -0.005)*	0.0003 (-0.02, 0.02)	-0.04 (-0.07, -0.01)*	-0.03 (-0.04, -0.01)*	0.04 (-0.02, 0.09)	0.03 (-0.06, 0.12)
ln organic Hg	-0.02 (-0.03, -0.003)*	-0.003 (-0.02, 0.01)	-0.02 (-0.04, -0.01)*	-0.01 (-0.02, -0.003)*	0.02 (-0.01, 0.06)	0.01 (-0.05, 0.08)
ln blood Cd	0.002 (-0.02, 0.02)	-0.01 (-0.03, 0.02)	-0.01 (-0.04, 0.01)	-0.01 (-0.03, 0.01)	0.05 (-0.07, 0.16)	0.09 (-0.02, 0.21)
ln urinary Cd	0.04 (0.005, 0.07)*	0.03 (-0.01, 0.07)	0.04 (-0.01, 0.08)	0.004 (-0.02, 0.03)	0.05 (-0.09, 0.19)	0.18 (-0.09, 0.44)

[a]: Adjusted for age, sex, race/ethnicity, creatinine adjusted urinary iodine, BMI value, and serum cotinine concentration

*: $p<0.05$

Supplemental Material, Table S4. Adjusted regression coefficients and 95% confidence intervals of cumulative exposure to lead, mercury, and cadmium in relation to thyroid hormones in adults[a]

Pb ≥1.39 μg/dL	Total Hg ≥0.88 μg/L	Cd ≥0.33 μg/L	$lnTT_4$	$lnFT_4$	$lnTT_3$	$lnFT_3$	lnTSH	lnTg
Yes	Yes	Yes	-0.02 (-0.06, 0.02)	0.01 (-0.02, 0.04)	-0.02 (-0.05, 0.01)	-0.01 (-0.03, 0.01)	-0.03 (-0.12, 0.06)	0.22 (0.04, 0.39)
Yes	Yes	No	-0.05 (-0.09, -0.004)*	-0.01 (-0.04, 0.02)	-0.04 (-0.07, -0.02)*	-0.01 (-0.03, 0.01)	-0.05 (-0.15, 0.04)	0.03 (-0.11, 0.18[illegible]
Yes	No	Yes	-0.0004 (-0.04, 0.04)	0.01 (-0.02, 0.04)	0.01 (-0.02, 0.04)	0.01 (-0.02, 0.04)	-0.10 (-0.22, 0.02)	0.24 (0.11, 0.37)
Yes	No	No	-0.02 (-0.06, 0.01)	0.001 (-0.03, 0.03)	0.01 (-0.02, 0.03)	0.01 (-0.02, 0.03)	0.004 (-0.11, 0.12)	0.07 (-0.07, 0.21[illegible]
No	Yes	Yes	-0.04 (-0.08, 0.002)	-0.01 (-0.04, 0.02)	-0.04 (-0.07, -0.01)*	-0.02 (-0.04, 0.01)	-0.05 (-0.21, 0.10)	0.17 (0.02, 0.32)
No	Yes	No	-0.04 (-0.07, -0.004)*	-0.02 (-0.04, 0.01)	-0.03 (-0.06, -0.01)*	-0.02 (-0.03, -0.002)*	0.02 (-0.08, 0.12)	0.11 (-0.02, 0.25[illegible]
No	No	Yes	-0.02 (-0.05, 0.01)	-0.003 (-0.03, 0.03)	0.002 (-0.03, 0.04)	-0.01 (-0.04, 0.02)	-0.03 (-0.19, 0.13)	0.19 (0.02, 0.36)
No	No	No	reference	reference	reference	reference	reference	reference

[a]: Adjusted for age, sex, race/ethnicity, creatinine adjusted urinary iodine, BMI value, and serum cotinine concentration

*: $p<0.05$
